# Death, reoperation, and late cardiopulmonary function after truncus repair

**DOI:** 10.1016/j.xjon.2023.02.010

**Published:** 2023-03-01

**Authors:** Takaya Hoashi, Kenta Imai, Naoki Okuda, Motoki Komori, Yoshikazu Ono, Kenichi Kurosaki, Hajime Ichikawa

**Affiliations:** a Department of Pediatric Cardiovascular Surgery, National Cerebral and Cardiovascular Center, Osaka, Japan; b Department of Pediatric Cardiology, National Cerebral and Cardiovascular Center, Osaka, Japan

**Keywords:** truncus arteriosus, truncal valve regurgitation, truncal root dilatation

## Abstract

**Objective:**

To identify the late surgical outcomes of truncus arteriosus.

**Methods:**

Fifty consecutive patients with truncus arteriosus who underwent surgery between 1978 and 2020 at our institute were enrolled in this retrospective, single institutional cohort study. The primary outcome was death and reoperation. The secondary outcome was late clinical status, including exercise capacity. The peak oxygen uptake was measured by a ramp-like progressive exercise test on a treadmill.

**Results:**

Nine patients underwent palliative surgery, which resulted in 2 deaths. Forty-eight patients went on to truncus arteriosus repair, including 17 neonates (35.4%). The median age and body weight at repair were 92.5 days (interquartile range, 10-272 days) and 3.85 kg (interquartile range, 2.9-6.5 kg), respectively. The survival rate at 30 years was 68.5%. Significant truncal valve regurgitation (*P* = .030) was a risk factor for survival. Survival rates were similar between in the early 25 and late 25 patients (*P* = .452). The freedom from death or reoperation rate at 15 years was 35.8%. Significant truncal valve regurgitation was a risk factor (*P* = .001). The mean follow-up period in hospital survivors was 15.4 ± 12 years (maximum, 43 years). The peak oxygen uptake, which was performed in 12 long-term survivors at a median duration from repair of 19.7 years (interquartile range, 16.8-30.9 years), was 70.2% of predicted normal (interquartile range, 64.5%-80.4%).

**Conclusions:**

Truncal valve regurgitation was a risk factor for both survival and reoperation, thus improvement of truncal valve surgery is essential for better life prognosis and quality of life. Slightly reduced exercise tolerance was common in long-term survivors.

## Graphical abstract

**Figure:**
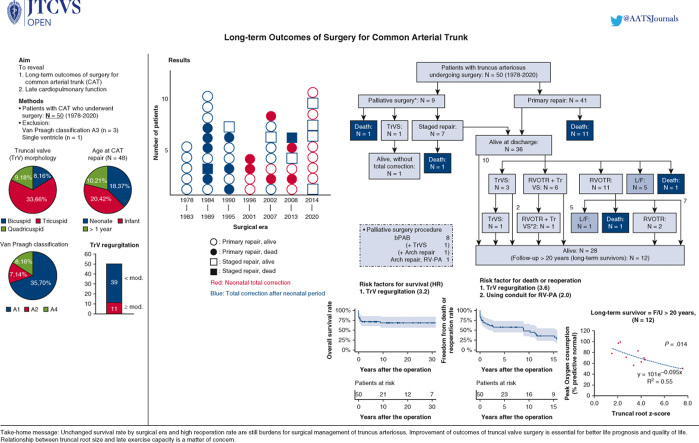

**Figure:**
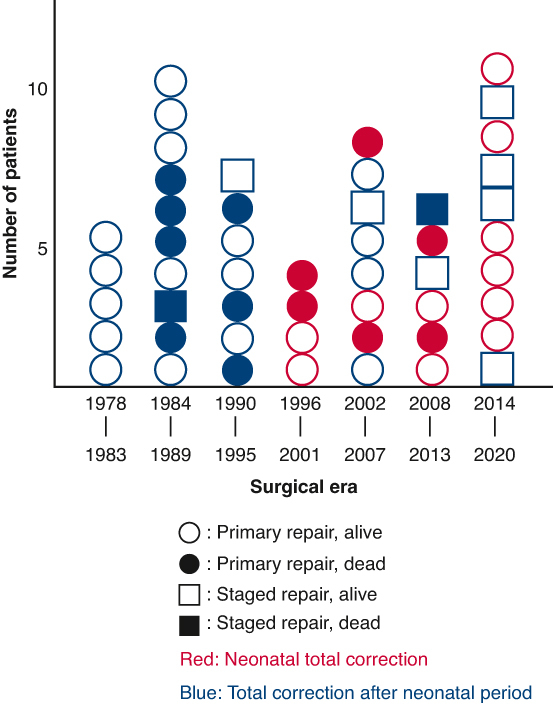
Prognoses of patients with truncus arteriosus after surgery by surgical era and strategy.

**Central Message**

The survival rate at 30 years was 68.5%. Although neonatal repair and early surgical era were not risk factors for survival, truncal valve regurgitation was a risk factor for survival and reoperation.

**Perspective**

The unchanged survival rate by surgical era and the high reoperation rate remain burdens in the surgical management of truncus arteriosus. Improvement in outcomes of truncal valve surgery is essential for better life prognosis and quality of life. Late survivors exhibited reduced exercise capacity. The relationship between truncal root size and late exercise capacity is a matter of concern.

Truncus arteriosus is among the most common conotruncal anomalies, and was reported to be present in approximately 6 per 100,000 live births.^1^ Since the first successful surgical experience was reported in 1968, the outcomes have improved and neonatal primary repair has been a standard approach.^2^, ^3^, ^4^, ^5^ Recent reports identified several risk factors for survival and unfavorable outcomes, including significant truncal valve (TrV) regurgitation, arch obstruction, abnormal coronary running, and prematurity.^6^, ^7^, ^8^, ^9^, ^10^, ^11^

A recent study showed that adult congenital heart disease patients showed unexpectedly reduced exercise intolerance, although they are apparently asymptomatic.^12^ Whereas reoperation for right ventricular outflow tract is unavoidable, life prognosis and quality of life were reported to be generally good after truncus arteriosus repair^13^, ^14^, ^15^, ^16^; nevertheless, the detailed late cardiopulmonary function has seldom been reported.^17^

This study reviewed single institutional long-term outcomes of surgery for truncus arteriosus to assess the mortality, reintervention, and current status of long-term survivors, including exercise capacity.

## Materials and Methods

### Patients

All 50 consecutive patients with truncus arteriosus who had undergone surgery between 1978 and 2020 were enrolled in this study (Table 1). Patients with Van Praagh classification type A3 (n = 3) or single ventricle (n = 1) were excluded because their surgical strategies were different. The National Cerebral and Cardiovascular Center Institutional Review Board approved this retrospective study (R19092, December 20, 2019), and opt-out consent was obtained instead of individual written informed consent.

**Table 1:** Patient characteristics

Characteristic	Result
Female	24 (48.0)
Van Praagh classification (n = 50, 100%)	
A1	35 (70.0)
A2	7 (14.0)
A4	8 (16.0)
TrV morphology (n = 50, 100%)	
Bicuspid	8 (16.0)
Tricuspid	33 (66.0)
Quadricuspid	9 (18.0)
TrV regurgitation ≥ moderate (n = 50, 100%)	11 (22.0)
22q11.2 deletion (1993-, n = 26, 52.0%)∗	4 (15.4)
Operative procedure: First palliative operation (n = 9, 18.0%)	
bPAB	8
Arch repair	2
TrV plasty	1
Right ventricle-to pulmonary artery conduit	1
Operative procedure: Second palliative operation (n = 3, 33.3%)	
bPAB	1
TrV repair, converted to replacement	1
Pulmonary artery plasty + SPS	1

Values are presented as n (%). *TrV*, Truncal valve; *bPAB*, bilateral pulmonary artery band; *SPS*, systemic-to-pulmonary shunt.

∗ Fluorescence in situ hybridization has been possible since 1993.

### Selection of Surgical Strategy and Timing of Truncus Arteriosus Repair

Throughout the study period, primary truncus arteriosus repair was the first-line treatment (Table 2). Repair was usually planned beyond the neonatal period, especially for low-birth-weight infants. Since 1996, primary repair has usually been performed during the neonatal period. Since 2005, palliative surgery, mainly pulmonary artery banding (either bilateral or main pulmonary trunk if present) concomitant with arch repair or truncal valve repair, has been selected for patients with aortic arch obstruction, significant TrV regurgitation, or for low-birth-weight infants. Before 2005, 2 patients had undergone palliative surgery before arrival.

**Table 2:** Operative characteristics: truncus arteriosus repair (n = 48)

Characteristic	Result
Primary repair	42 (87.5)
Staged repair	6 (22.5)
Age (d)	93 (10-272)
Neonate	18 (37.5)
Body weight (kg)	3.85 (2.9-6.5)
Major concomitant procedure(s) (n = 17, 35.4%)	
Truncal valve surgery	12 (25.0)
Arch repair	5 (10.4)
ECMO procedure	5 (10.4)
Materials for RVOTR (n = 48, 100%)	
Posterior AP patch, anterior patch with monocusp (1998∼2012)	14 (29.2)
AP roll with pericardial trileaflet (1986∼1992)	10 (20.8)
Contegra bovine jugular vein conduit (2013∼)	8 (16.7)
Ionescu-Shiley (1981∼1985)	4 (8.3)
Posterior direct, anterior patch with monocusp (1993∼1997)	4 (8.3)
Hancock valved conduit (∼1980)	3 (6.3)
Others	5 (10.4)
Aortic crossclamp time (min) (n = 40, 83.3%)	74.5 (48-93)
Cardiopulmonary bypass time (min) (n = 40, 83.3%)	174 (127-232)

Values are presented as n (%) or median (interquartile range). *ECMO*, Extracorporeal membrane oxygenation; *RVOTR*, right ventricular outflow tract reconstruction; *AP*, autopericardium.

### Truncus Arteriosus Repair

Truncus repair, which consists of the division of pulmonary artery from truncal root, closure of the ventricular septal defect, and creation of right ventricle-to-pulmonary artery continuity, was carried out by standard technique according to previous reports^2^, ^3^, ^4^, ^5^ (Video 1). Due to the long study period, various types of right ventricular outflow tract reconstruction procedures were selected (Table 2). Our method of palliative banding for symptomatic cases has been previously reported.^18^ For arch repair, end-to-end direct anastomosis was a standard approach. Graft interpose was selected in an older child. For more complex cases, Norwood-like arch reconstruction was selected.^19^ For TrV repair, complete closure or plication of regurgitant commissure(s) was the standard approach. Thickened leaflets and raphe were occasionally sliced. In a recent 2.5-kg infant, cusp reconstruction was attempted using glutaraldehyde-treated autologous pericardium.^20^

### Study Design and Statistical Analysis

This was a retrospective, single-center cohort study. The primary outcome was death and reoperation. The secondary outcome was late clinical status, including exercise capacity. The evaluated variables were as follows:

• Overall outcomes: Prognoses of patients after surgery by surgical era and strategy, therapeutic algorithm and outcomes of individual patients, overall survival and freedom from death and reoperation rates by the Kaplan-Meier method, and an analysis of risk factors by the Cox proportional hazards model;
• Current status of long-term survivors; and
• Factors affecting late peak oxygen uptake (pvo_2_) by a nonlinear regression model. Possible risk factors were picked up from previous reports such as truncal root diameter *z* score, total number of sternotomies, and tricuspid valve regurgitation pressure gradient.^17^^,^^21^

A ramp-like progressive exercise test on a treadmill was employed to measure ventilatory threshold and pvo_2_. Predicted pvo_2_ was calculated from the institutional original formulae.^22^ Truncal root diameter was converted to *z* score based on normal aortic root data for adults.^23^

Continuous variables are presented as median (interquartile range [IQR]). Data in this study were analyzed using the SPSS statistical software platform (SPSS Inc). Normal distributions of variables were confirmed by the Shapiro-Wilk test. Possible risk factors for mortality were first identified by univariable analysis, and variables with *P* values < .20 were used in the multivariate Cox proportional hazards model.

## Results

### Patient Characteristics

Twenty-eight patients (56.0%) were women (Table 1). TrVs were bicuspid in 8 patients (16.0%), tricuspid in 33 patients (66.0%), and quadricuspid in 9 patients (18.0%). Significant TrV regurgitation was present in 11 patients (22.0%). Aortic arch obstruction coexisted in 8 patients (16.0%). The 22q11.2 deletion had been detected in 4 of 26 patients (15.4%) since 1994. Palliative operations were performed in 9 patients (18.0%).

Forty-eight patients underwent truncus arteriosus repair, including 18 neonates (37.5%) (Table 2). Median age at repair was 93 days (IQR, 10-272). Median body weight at repair was 3.85 kg (IQR, 2.9-6.5 kg). The major concomitant procedures were truncal valve surgery in 12 patients (25.0%) and arch repair in 5 patients (10.4%). Due to the long study period, various procedures for right ventricular outflow tract reconstruction were adopted. Conduit was used in 29 of 48 patients (60.4%) at right ventricular outflow tract reconstruction. The median aortic crossclamp time and cardiopulmonary bypass time were 74.5 minutes (IQR, 48-93 minutes) and 174 minutes (IQR, 127-232 minutes), respectively.

### Overall Outcomes

Follow-up was conducted between February 2019 and January 2022. Although late survivors were routinely followed annually, the recent COVID-19 pandemic did not allow some patients to come to our outpatient clinic between February 2020 and December 2021. Thus, we reviewed the most recent 3 years' outpatient clinic records. At this time, 29 patients were alive, 15 patients were dead, and 6 patients did not come to the clinic (but were not known to be dead). Timing and cause(s) of death are summarized in Table E1.

**Figure 1:**
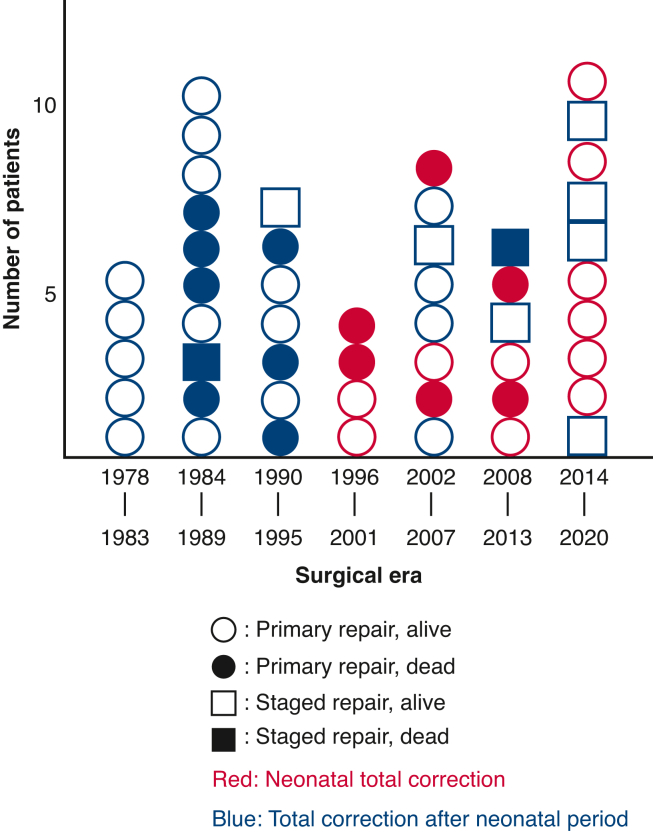
Prognoses of patients with truncus arteriosus undergoing surgery by surgical era and strategy. *Circles* represent primary repair. *Squares* represent staged repair. *Open symbols* represented survivors. *Closed symbols* represent mortalities. Neonatal total correction is in *red*. Total correction after the neonatal period is in *blue*.

**Figure 2:**
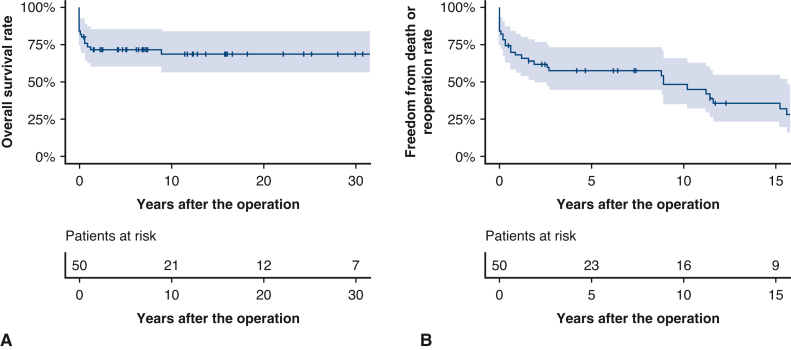
(A) Overall survival and (B) freedom from death or reoperation rates by Kaplan-Meier method. *Bars* represented 95% CI.

Number of patients by surgical era as well as selected strategy and prognostic outcomes are shown in Figure 1. The mean follow-up period in hospital survivors was 15.4 ± 12 years (maximum, 43 years). Overall survival rates at 10, 20, and 30 years were 68.8% each (Figure 2, *A*). The number at risk at 10, 20, and 30 years was 21, 12, and 7 patients, respectively. The univariable Cox proportional hazards model identified TrV regurgitation (hazard ratio [HR], 3.586; 95% CI, 1.120-9.215; *P* = .030) as a risk factor for survival (Table E2). Survival rates were similar between the early 25 and late 25 patients (*P* = .43).

**Figure 3:**
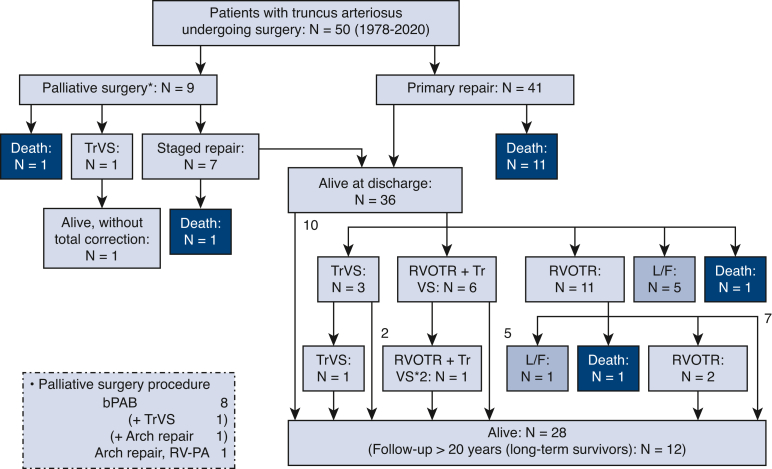
Therapeutic algorithm and outcomes of 50 individual patients with truncus arteriosus undergoing surgery. *TrVS*, Truncal valve surgery; *RVOTR*, right ventricular outflow tract reconstruction; *L/F*, lost to follow-up; *bPAB*, bilateral pulmonary artery band; *RV-PA*, right ventricle to pulmonary artery.

Therapeutic algorithm and outcomes of individual patients are shown in Figure 3. The freedom from death or reoperation rates at 5, 10, and 15 years were 48.8%, 35.3%, and 26.0%, respectively (Figure 2, *B*). The number at risk at 5, 10, and 15 years was 23, 16, and 9 patients, respectively. In total, 20 patients underwent 25 reoperations (Table E3). Three patients underwent 2 reoperations, and 1 patient underwent 3 reoperations (Figure 3). There were 21 reoperations and 7 catheter interventions for the right ventricular outflow tract to the pulmonary artery. Reoperations included conduit size-up in 10 cases, trans-annular patching in 6 cases, and branch pulmonary artery plasty in 1 case. No patients required stent implantation. Ten patients underwent various types of TrV repair, which resulted in 5 deaths and 2 subsequent replacements, and the remaining 3 patients were alive without additional surgery for TrV. Including the above-mentioned 2 patients, 10 patients required TrV replacement (mechanical valve in 9 patients, pulmonary homograft in 1 patient). Concomitant procedures at TrV replacement were aortoventriculoplasty (ie, Konno) and root replacement (ie, Bentall-type) in 1 in each. No patients experienced dissection or rupture of the truncal root or ascending aorta.

The multivariable Cox proportional hazards model showed that TrV regurgitation (HR, 4.395; 95% CI, 1.815-10.642; *P* = .001) and right ventricular outflow tract reconstruction using conduit (HR, 2.217; 95% CI, 1.017-4.831; *P* = .045) were risk factors for death or reoperation (Table E2).

### Current Status of Long-term Survivors

Twelve patients who have survived for more than 20 years after truncus arteriosus repair were defined as long-term survivors (Table 3). The median follow-up duration was 31.5 years (IQR, 27.3-34.5 years). Of all 12 long-term survivors, 10 patients have undergone redo of right ventricular outflow tract procedures and 5 patients have undergone TrV replacement.

**Table 3:** Late outcomes in long-term survivors (n = 12)

Outcome	Result
Follow-up duration (y)	31.5 (27.3-34.5)
Post-redo RVOTR	10 (83.3)
Post-TrV replacement	5 (41.7)
NYHA functional status class	
I	11 (91.7)
II	1 (8.3)
Medication	
Yes	6 (50.0)
No	6 (50.0)
Serum BNP (pg/mL) (n = 12, 100%)	19.2 (14.8-37.0)
Cardiopulmonary exercise testing (n = 12, 100%)	
Duration from truncus arteriosus repair (y)	19.7 (16.8-30.9)
Anaerobic threshold (% of predictive normal)	83.4% (72.1-91.0)
Peak oxygen uptake (% of predictive normal)	70.2% (64.5-80.4)
Transthoracic echocardiogram (n = 12, 100%)	
Duration from operation (y)	30.3 (24.6-34.5)
Truncal root diameter (mm)	40.1 (34.0-42.0)
*z*-score	3.4 (2.4-4.1)
TrV regurgitation (n = 7) < Mild	4 (57.1)
Mild	3 (42.9)
MR < mild	10 (83.3)
Mild	1 (8.3)
Moderate	1 (8.3)
LVEDd (mm)	43.5 (40.5-47.3)
LVEDd (% of predictive normal)	90.0 (89.8-98.0)
LVEF (%)	64.5 (58.0-66.5)
RVEDd (mm)	32.5 (26.9-38.8)
TRPG (mm Hg)	31.0 (27.0-42.5)

Values are presented as median (interquartile range) or n (%). *RVOTR*, Right ventricular outflow tract reconstruction; *TrV*, truncal valve; *NYHA*, New York Heart Association functional classification; *BNP*, brain natriuretic peptide; *MR*, mitral regurgitation; *LVEDd*, left ventricular end-diastolic diameter; *LVEF*, left ventricular ejection fraction; *RVEDd*, right ventricular end-diastolic diameter; *TRPG*, tricuspid regurgitation pressure gradient.

All 12 patients aged 18 years or older routinely underwent cardiopulmonary exercise testing, except for 1 patient who could not walk normally. Cardiopulmonary exercise testing was performed at a median duration from truncus repair of 19.7 years (IQR, 16.8-30.9 years). The anaerobic threshold and pvo_2_ was 83.4% of predicted normal (IQR, 72.1%-91.0%) and 70.2% of predicted normal (IQR, 64.5%-80.4%), respectively. The latest transthoracic echocardiograms showed that the median left ventricular ejection fraction was 64.5% (IQR, 58.0%-66.5%). Moderate or greater mitral regurgitation was observed in 1 patient. Median truncal root *z* score was 3.4 (IQR, 2.4-4.1).

### Factors Influencing Late pvo_2_

**Figure 4:**
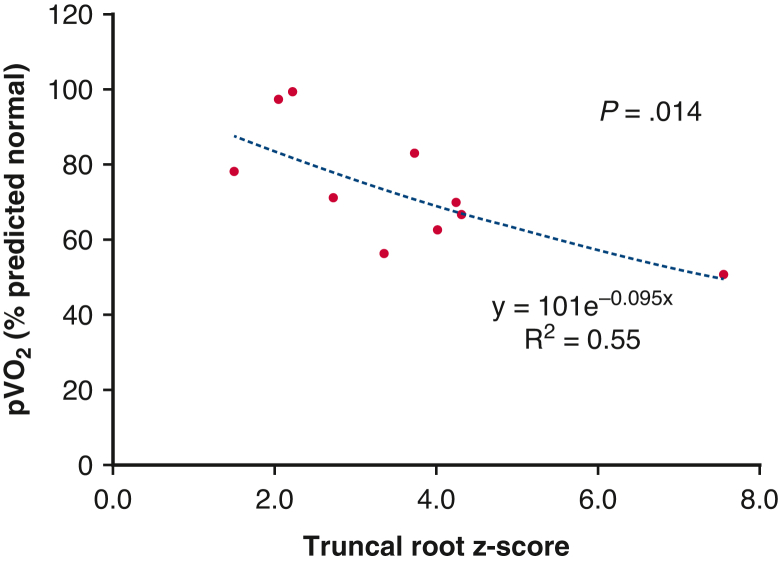
Relationship between the truncal root *z* score and peak oxygen uptake (*p**vo*_*2*_) in late survivors (n = 10).

Truncal root diameter z score was significantly and exponentially correlated with pvo_2_ (y = 101e-0.095x; *R*^2^ = 0.55; *P* = .014) (Figure 4). One patient with a truncal root diameter of 7.56 subsequently underwent a Bentall operation. The total number of thoracotomy/sternotomy, tricuspid valve regurgitation pressure gradient, age at repair of truncus arteriosus, and anaerobic threshold was not correlated (Table E4).

## Discussion

**Figure 5:**
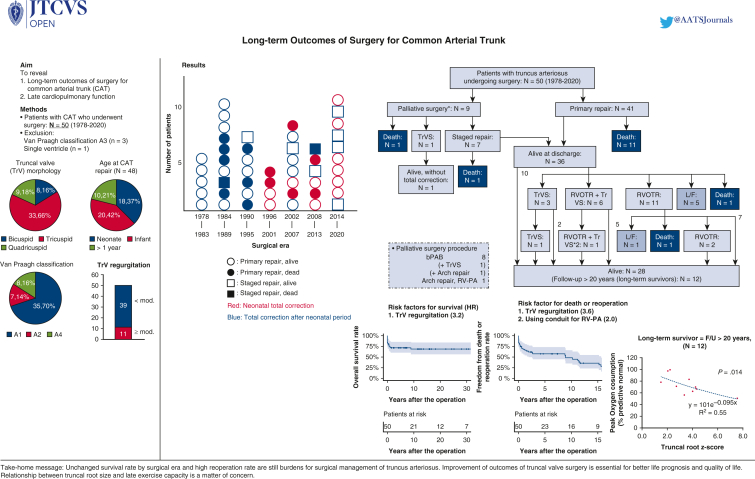
Truncal valve (*TrV*) regurgitation was a risk factor for both survival and reoperation. As such, improvement of TrV surgery is essential for better life prognosis and quality of life. Staged repair should be selected for patients with Van Praagh A4. Late exercise capacity was slightly reduced and correlated to the size of truncal root. *RVOTR*, Right ventricular outflow tract reconstruction; *L/F*, lost to follow-up; *bPAB*, bilateral pulmonary artery band; *RV-PA*, right ventricle to pulmonary artery; *F/U*, follow-up.

Consistent with previous studies, this study demonstrated that TrV regurgitation was found to be a risk factor for both survival and reoperation^6^, ^7^, ^8^^,^^10^ (Figure 5). The repair technique adopted in this study was mainly bicuspidization or tricuspidization of the tricuspid or quadricuspid valve by commissure(s) closure, and plication of the regurgitant commissure(s), with or without thickened leaflet slicing. However, to date, the therapeutic effect is limited. Because the poor survival and valve durability after neonatal truncal valve repair are recognized, the development of a new technique (neocuspidization^24^), innovation of small-sized valve prostheses, and improvement of small-sized homograft availability are essential to improving long-term outcomes.

The unchanged survival rates between the early and late study period should not be misunderstood. Because this study was based on an institutional surgical database, we cannot take account of patients with truncus arteriosus who died without any interventional medical treatment during the same study period. Nevertheless, more patients underwent neonatal truncus repair in the late study period, as shown in Figure 1, which means that only patients who could survive beyond the neonatal (or early infantile) period without medical treatment were eligible for surgical repair during the early study period. As shown in Figure 1, another thing to consider is that staged repair tended to be selected in more patients in the late study period, which may indicate that patients with more complex truncus arteriosus such as that coexisting with arch obstruction, TrV regurgitation, and prematurity were included in the late study period. Owing to this treatment change, arch obstruction and low birth weight (<2500 g) were no longer risk factors for survival; therefore, the management of TrV regurgitation was again the key to better life prognosis, as described above.

As shown in Figure 2, reoperation after truncus repair was frequent, especially within 3 years after the repair, mainly for TrV regurgitation, and around 10 years after the repair, mainly for conduit exchange and TrV replacement. Whereas right ventricular outflow tract reconstruction without conduit such as the Barbero-Marcial procedure, direct pulmonary artery anastomosis, or our original method appear to be good options to prevent frequent reoperations, the early development of pulmonary insufficiency is unavoidable.^6^^,^^25^^,^^26^ Whereas a recent report has shown that small conduit size was a risk for both mortality and early reoperation, the placement of a larger conduit in neonatal primary repair involves an excessively large right ventriculotomy, which can sometimes critically impair right ventricle function.^27^ Large conduit placement also carries a risk of conduit compression from the chest wall, which may require early conduit exchange. Together with modification of the existing technique, innovation of the valved conduit with growth potential is expected.^28^

Dilation of the aortic root is a specific phenomenon in patients with conotruncal anomaly. Although the inherent structural abnormality of the aortic wall was considered to be the main etiology, acquired hemodynamic overload was suspected to advance it secondarily.^29^ A previous study demonstrated that, in patients with truncus arteriosus, the degree of truncal root dilatation was significantly associated with the severity of TrV regurgitation.^30^ It is not surprising that shear stress to the truncal root wall by to-and-fro regurgitant blood flow across the TrV accelerates dilation of the truncal root. In contrast, a recent study showed that truncal root dilation before truncus arteriosus repair was related to the development of late TrV regurgitation.^31^ This study indicates that patients who had an intrinsically more vulnerable truncal root wall are likely to experience late, significant TrV regurgitation. Unfortunately, our study did not show a statistically significant relationship between truncal root size and the degree of TrV regurgitation in late survivors because the number of late survivors was small and approximately half of patients had already undergone TrV replacement before cardiopulmonary exercise testing. Instead, this study revealed that truncal root *z* score was negatively correlated with exercise capacity. Recently, the size and stiffness of the ascending aorta have been shown to be significantly correlated with exercise intolerance in patients with repaired congenital heart disease.^21^ Excessive caliber-size change between the dilated truncal root and the normal-sized aortic arch to the descending aorta, reduced compliance of the truncal root wall, and possible coexisting TrV disease may inhibit the increase of cardiac output in response to exercise, thereby causing exercise intolerance.

The dilated aortic root in patients with conotruncal anomaly is believed to be rarely ruptured or dissected,^31^ so indication for surgery is based on adult criteria.^32^ As described above, TrV regurgitation causes or is caused by truncal root dilatation, TrV regurgitation is a risk factor in death and reoperation, and truncal root dilatation is related to late exercise intolerance. Against this background, more preventive truncal valve and truncal root surgery should be justified.

### Study Limitations

This study is a more than 40-year-long retrospective series consisting of a small number of patients. Pre- and perioperative data, such as diameter of the truncal root, TrV stenosis, or location of coronary artery orifices, were often missing. Surgical strategies, especially materials for reconstruction of the right ventricle to pulmonary artery continuity, have changed. Spirometry was performed in only half of the long-term survivors, so the relationship between restrictive respiratory dysfunction and pvo_2_ could not be examined.

## Conclusions

The unchanged survival rate by surgical era and the high reoperation rate remain burdens in the surgical management of truncus arteriosus. Although neonatal repair and early surgical era were not risk factors for survival, TrV regurgitation was a risk factor for survival and reoperation. Thus, improvement of TrV surgery is essential for better life prognosis and quality of life. Late survivors showed reduced exercise capacity, and the relationship between truncal root size and late exercise capacity is a matter of concern.

### Conflict of Interest Statement

The authors reported no conflicts of interest.

The *Journal* policy requires editors and reviewers to disclose conflicts of interest and to decline handling or reviewing manuscripts for which they have a conflict of interest. The editors and reviewers of this article have no conflicts of interest.

## Figures and Tables

**Table E1:** Timing and cause(s) of death (n = 15)

Patient No.	Duration from surgery to death (d)	Reason for death
1	4	Unknown
2	1	Right heart failure
3	3238	Massive multiple cerebral infarction 1 d after redo RVOTR
4	1	PH, hypoxia
5	343	Infection after redo RVOTR
6	10	Biventricular failure (with LVOTO)
7	421	Sudden death in home
8	1	Left heart failure (derived from Barbero-Marcial repair?)
9	12	Biventricular failure
10	7	PH, hypoxia
11	220 (before discharge)	Chronic left heart failure (severe TrV regurgiation), venous obstruction, infection
12	226 (before discharge)	PH, rihgt heart failure
13	9	PH
14	66	Right heart failure
15	40	Left heart failure (severe TrV regurgitation)

*RVOTR*, Right ventricular outflow tract reconstruction; *PH*, pulmonary hypertension; *LVOTO*, left ventricular outflow tract obstruction; *TrV*, truncal valve.

**Table E2:** Cox proposal hazard model for survival and reoperation

Variable	*P* value	Hazard ratio (95% CI)
Survival, univariable		
TrV regurgitation (Yes, n = 11, No, n = 39)	.030	3.213 (1.120-9.215)
Birth weight <2500 g (Yes, n = 11, No, n = 25, unknown, n = 14)	.054	4.390 (0.976-19.741)
Concomitant arch repair (Yes, n = 5, No, n = 43, without repair, n = 2)	.132	2.682 (0.742-9.685)
Arch obstruction (Yes, n = 8, No, n = 42)	.202	2.112 (0.670-6.660)
Surgical era [Early (−1999, n = 25) vs Late (2000-, n = 25)]	.452	0.672 (0.239-1.894)
Staged repair (Yes, n = 9, No, n = 41)	.678	0.707 (0.159-3.135)
Neonatal repair (Yes, n = 18, No, n = 30, without repair, n = 2)	.752	1.181 (0.420-3.321)
Reoperation, univariable		
TrV regurgitation (Yes, n = 11, No, n = 39)	.003	3.586 (1.566-8.210)
RVOT procedure, conduit (Yes, n = 29, No, n = 19, without repair, n = 2)	.085	1.957 (0.911-4.206)
Staged repair (Yes, n = 9, No, n = 41)	.286	0.563 (0.196-1.617)
Birth weight < 2500g (Yes, n = 11, No, n = 25, unknown, n = 14)	.508	1.356 (0.550-3.342)
Neonatal repair (Yes, n = 18, No, n = 30, without repair, n = 2)	.588	1.222 (0.592-2.523)
Concomitant arch repair (Yes, n = 5, No, n = 43, without repair, n = 2)	.597	1.385 (0.414-4.634)
Surgical era [Early (−1999, n = 25) vs Late (2000-, n = 25)]	.662	0.853 (0.417-1.743)
Arch obstruction (Yes, n = 8, No, n = 42)	.905	0.938 (0.327-2.692)
Reoperation, multivariable		
TrV regurgitation	.001	4.395 (1.815-10.642)
RVOT procedure, conduit	.045	2.217 (1.017-4.831)

*CI*, Cardiac index; *TrV*, truncal valve; *RVOTR*, right ventricular outflow tract.

**Table E3:** Procedures for reoperations

Procedure	Total No.
Reconstruction of right ventricular outflow tract to pulmonary artery	21
Truncal valve repair/replacement	13
Tricuspid valve repair	2
Diaphragmatic plication	2
Revision of VSD closure	2
Repair of unroofed coronary sinus	1
Repair of partial anomalous pulmonary venous return	1
Permanent pacemaker implantation	1

*VSD*, Ventricular septal defect.

**Table E4:** Logarithmic approximation of factors correlating with peak oxygen uptake long after the repair of truncus arteriosus

Variable	*R*^2^	*P* value
Truncal root *z* score (n = 10)	0.55	.014
Age at the repair of truncus arteriosus (days-old, n = 11)	0.301	.081
Anaerobic threshold (% of predicted normal, n = 11)	0.203	.191
No. of sternotomy (times, n = 11)	0.176	.199
Peak systolic tricuspid pressure gradient (mm Hg, n = 10)	0.105	.33

## Supplementary Data

Video 1 The patient was an 8-day-old neonate with truncus arteriosus type I. Through median sternotomy, cardiopulmonary bypass was established and the aorta was then cross-clamped. The pulmonary arterial trunk was divided from the common arterial trunk, and a right ventriculotomy was made. The ventricular septal defect was closed with an expanded polytetrafluorethylene patch via the right ventriculotomy. The patent foramen ovale was closed primarily. After aortic unclamping, 12 mm of bovine jugular vein conduit was interposed between the branch pulmonary artery and the right ventriculotomy. Video available at: https://www.jtcvs.org/article/S2666-2736(23)00037-2/fulltext.
