# Supplementary figures and images for: Death, reoperation, and late cardiopulmonary function after truncus repair
Source: JTCVS Open. 2023 Mar 1;14:407–16. doi: 10.1016/j.xjon.2023.02.010 (PMC10328806; doi:10.1016/j.xjon.2023.02.010)

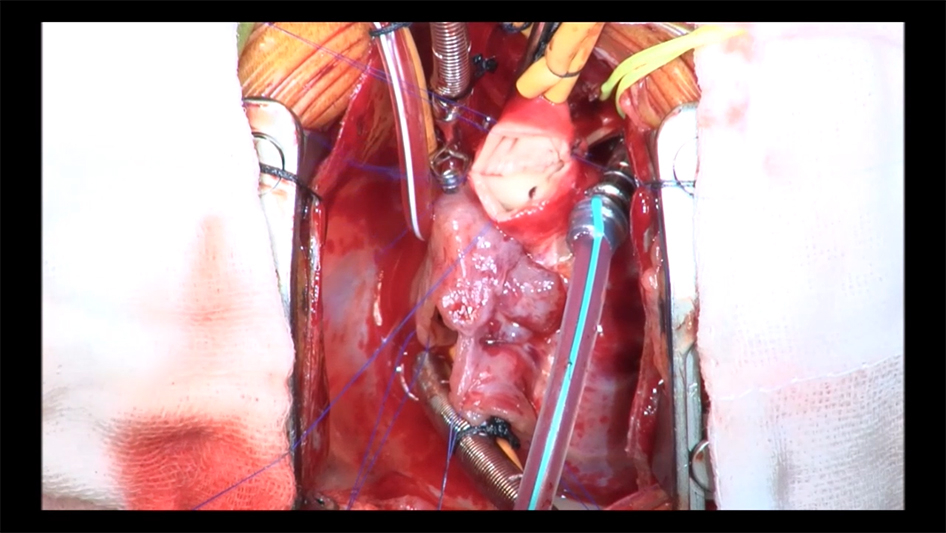

Supplement: Video 1 — The patient was an 8-day-old neonate with truncus arteriosus type I. Through median sternotomy, cardiopulmonary bypass was established and the aorta was then cross-clamped. The pulmonary arterial trunk was divided from the common arterial trunk, and a right ventriculotomy was made. The ventricular septal defect was closed with an expanded polytetrafluorethylene patch via the right ventriculotomy. The patent foramen ovale was closed primarily. After aortic unclamping, 12 mm of bovine jugular vein conduit was interposed between the branch pulmonary artery and the right ventriculotomy. Video available at: https://www.jtcvs.org/article/S2666-2736(23)00037-2/fulltext. [file fx3.jpg]
